# Development and field test of the child and adolescent sleep checklist for parents of community junior high school students

**DOI:** 10.3389/frcha.2025.1644128

**Published:** 2025-08-12

**Authors:** Kentaro Kawabe, Saori Inoue, Yu Matsumoto, Maya Kusunoki, Shu-Ichi Ueno, Yasunori Oka, Fumie Horiuchi

**Affiliations:** ^1^ Department of Child Psychiatry, Ehime University Graduate School of Medicine, Toon, Japan; ^2^ Department of Neuropsychiatry, Molecules and Function, Ehime University Graduate School of Medicine, Toon, Japan; ^3^ Center for Sleep Medicine, Ehime University Hospital, Toon, Japan

**Keywords:** sleep disorders, sleep checklist, CASC, parent-reported, validation, factor analysis, early adolescents, Japanese

## Abstract

**Aim:**

Children and adolescents get fewer than the recommended hours of sleep. The Child and Adolescent Sleep Checklist for parents (CASC-P) was designed to identify sleep habits and screen for sleep problems in junior high school students in Japan. This study aimed to validate the Japanese version of the CASC-P for junior high school students and determine its internal consistency.

**Methods:**

We used confirmatory factor analysis (CFA) and Cronbach's α to validate the scale and examine reliability. The analysis involved 218 parents of students aged 12–15 years.

**Results:**

Cronbach's α for the overall scale was 0.771. The prevalence of sleep problems was 15.6%. Factorial construct validity was assessed using the four-factor model used in the original CASC-P. Almost all items loaded meaningfully on their designated factors, and standardized factor-loading values ranged from 0.278 to 0.878 (except for items 1, 2, 9, 16, and 21).

**Conclusion:**

: The CASC-P is a suitable questionnaire for assessing parents’ perspectives on adolescent sleep behavior.

## Introduction

Sleep is a crucial component of childhood and adolescent development and health. Similar to healthy diet and exercise, sleep is critical for children to stay healthy, grow, learn, perform well in school, and function at their best (1). Late sleep timing, chronic sleep issues, and wakefulness disorders are associated with poorer health outcomes in children and adolescents, including physical and mental health, cognitive function or academic performance, eating behaviors, and risk of other mental health issues (2–4). Teenagers aged 14–17 years require 8–10 h of sleep per day (5). However, children and adolescents get fewer than the recommended sleep hours (6).

Many internal and external factors have been identified as detrimental to children's sleep. A clinical review reported that tobacco, digital media, evening lights, a negative family environment, and caffeine are detrimental to sleep (7). Sleep disorders are usually assessed using objective measures such as polysomnography (PSG), actigraphy, and sleep logs. Although PSG and actigraphy are useful tools for physiologically assessing sleep quality and quantity, they are highly specialized and expensive, whereas sleep logs and questionnaires are inexpensive and simple subjective measures for detecting sleep duration and sleep problems (8). Spruyt et al. conducted a comprehensive review and suggested that many of the tools used for children had not undergone careful and methodical psychometric evaluations (9). The Child and Adolescent Sleep Checklist for Parents (CASC-P) was designed to identify sleep habits and screen for sleep problems in preschoolers, elementary school children, and junior high school students in Japan. However, few Japanese sleep questionnaires have been internationally standardized, and the CASC-P has not yet been validated. This study aimed to validate the Japanese version of the CASC-P for junior high school students and determine its internal consistency.

## Methods

This cross-sectional survey was conducted in Kumakogen Town (population: 6,700), a rural region of Ehime Prefecture (population: 1,290,000) in southwest Japan. The participants were parents with children enrolled in three different public junior high schools in Kumakogen Town. All parents were recruited based on whether their children were attending school. In September 2012, questionnaires were distributed to the parents by their children. The parents completed the questionnaires at home.

### Ethical considerations

This study was approved by the Institutional Review Board of Ehime University Graduate School of Medicine (IRB No. 1206009) and the School Board of Kumakogen Town. Parents were informed of the study by letter and were encouraged to contact the school for any clarification.

### Instruments

Participants completed a demographic sheet and the CASC-P. The demographic sheet included basic information such as age, gender, and their children's grades.

### Sleep habits and sleep-related problems

Sleep habits and problems were assessed using the CASC-P. This checklist was designed to identify sleep habits and screen for sleep problems in preschoolers, elementary school children, and high school students. The CASC was developed for both clinical and research purposes (10, 11). The language options available for the original version were English and Japanese (12). A Chinese translated version of the CASC-P was validated and used in Taiwan (13, 14). The parental version is used for all age groups (ages 3–18) and is completed by parents. The CASC-P consists of two sections: 12 questions on sleep habits and a 24-item checklist that addresses sleep-related problems. The first section includes questions about total sleep time, bedtime, wake time on weekdays and weekends, nap frequency, and nap duration over the previous week; this section is not part of the questionnaire scoring. Regarding the second section, particpants were asked to respond using a 4-point frequency scale in which 0 indicated “never/unknown,” 1 indicated “occasionally” (0–1 day per week), 2 indicated “sometimes” (2–4 days per week), and 3 indicated “always” (5–7 days per week), or they responded “I don't know.” This results in an overall score ranging from 0 to 72, with scores greater than 18 indicating that the child likely has a sleep problem (10, 12). The CASC-P scores were subdivided into four categories: bedtime problems (Q1–Q6), sleep breathing and unstable sleep (Q7–Q12), parasomnia and sleep movement (Q13–Q18), and daytime problems (Q19–Q24) (10, 12).

### Statistical analysis

The results were expressed as the mean ± standard deviation (SD) for continuous variables and as numbers and percentages for categorical variables. Descriptive statistics were used to describe the distribution of student characteristics. Chi-square tests were employed to compare the ratio of students by gender, and the Mann–Whitney *U* test was employed to compare the total CASC-P score by grade. We used confirmatory factor analysis (CFA) for reliability and validity. The Cronbach's alphas for the overall and subscale scores were estimated to determine internal consistency. The Kaiser-Meyer-Olkin (KMO) statistic and Bartlett's test were estimated. The value of KMO equal to or greater than 0.5 and with significant values is less than 0.05 means the factor analysis is useful. All tests were two-sided and the significance level was set at 5%. All data were analyzed using the SPSS Statistics software and AMOS (version 28.0; IBM Corp., Armonk, NY, USA) for Windows.

## Results

**Figure 1:**
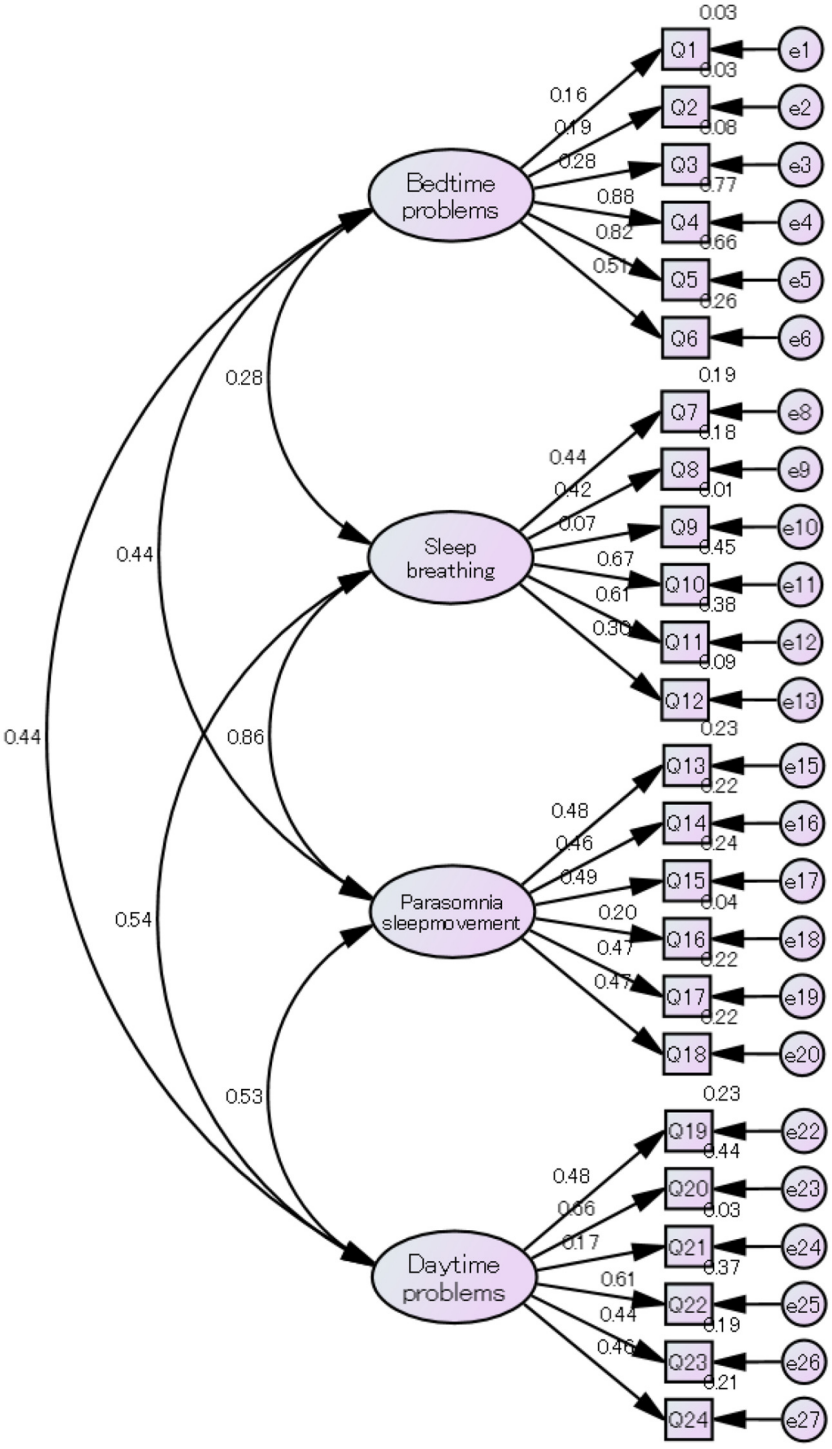
Confirmatory factor analysis of the child and adolescent sleep checklist for parents.

A total of 218 parents (108 male, 110 female) of students in grades 7–9, aged 12–15 years, were included after excluding two incomplete questionnaires. In the present sample (*N* = 218), Cronbach's α for the overall scale was 0.771, and the subscale coefficients were 0.592 (bedtime problems), 0.561 (sleep breathing and unstable sleep), 0.550 (parasomnia and sleep movement), and 0.626 (daytime problems). The results of the ratio of students by gender and the total CASC-P score by grade are presented in Table 1. There were no significant differences according to grade. The average CASC-P scores are presented in Table 2. According to the CASC-P cut-off score, the prevalence of sleep problems was 15.6%. Factorial construct validity was assessed using the four-factor model used in the original CASC-P. The sample size of 218 satisfied the estimated size requirement (15). The value of KMO is 0.71, and the Bartlett's test of sphericity (Chi-square = 1,181.8; *P* < 0.001). Multiple fit indices were used for the CFA. The selected indices were the chi-square statistic (CMIN/DF), standardized root mean square residual (SRMR), adjusted goodness of fit index (AGFI), comparative fit index (CFI), and root mean square error of the approximation (RMSEA). As shown in Table 3, AGFI and CFI did not indicate a good fit for the data (16). For the CASC-P, nearly all items loaded meaningfully onto their designated factors, with standardized factor loadings ranging from 0.278 to 0.878. Exceptions included items 1 (0.159), 2 (0.186), 9 (0.072), 16 (0.199), and 21 (0.170). Detailed results are shown in Figure 1 and the supplementary materials in Supplementary Table 1A.

**Table 1:** Demographic characteristics of the students (*N* = 218).

Item	Total	Seventh grade	Eighth grade	Ninth grade	*P* value
Number, *n*	218	77	64	77	
Sex (male), *n* (%)	108 (49.5)	42 (54.5)	31 (48.4)	35 (45.4)	0.52
Total CASC-P score	11.3 ± 6.9	11.1 ± 6.9	11.8 ± 7.2	11.2 ± 6.6	0.89
Total ≥18, *n* (%)	34 (15.6)	11 (14.3)	9 (14.1)	14 (18.2)	0.74

**Table 2:** Average score on the CASC-P.

Categories	Items	Score
Bedtime problems	1. I drink a caffeinated beverage ≤3h before going to bed	1.0 ± 1.1
	2. I play video games, surf the Internet, or send texts ≤1h before going to bed	1.5 ± 1.2
	3. I avoid going to bed even though it is time to go to sleep	1.0 ± 1.0
	4. I feel anxious or afraid when it is time to go to sleep	0.1 ± 0.5
	5. I have trouble falling asleep when I am by myself	0.2 ± 0.6
	6. Before I fall asleep, my legs feel uncomfortable, as if I cannot hold them still	0.1 ± 0.5
Sleep breathing and unstable sleep	7. I snore	0.6 ± 0.8
	8. My breath sounds as if it is getting caught in my throat	0.1 ± 0.4
	9. I stop breathing while I sleep	0.1 ± 0.4
	10. I toss and turn, or change positions often while I sleep	0.8 ± 0.9
	11. I talk in my sleep	0.6 ± 0.3
	12. I cry out in my sleep and wake up during the night	0.1 ± 0.3
Parasomnia and sleep movement	13. I have scary dreams or cry out during nightmares	0.2 ± 0.4
	14. I sleepwalk	0.1 ± 0.2
	15. My legs twitch while I sleep	0.3 ± 0.5
	16. I urinate in my sleep	0.0 ± 0.1
	17. I grind my teeth while I sleep	0.3 ± 0.6
	18. I sweat excessively while I sleep	0.4 ± 0.7
Daytime problems	19. The amount of sleep I get varies each night	0.6 ± 0.7
	20. I feel tired or groggy when I wake up in the morning	1.2 ± 1.0
	21. I skip breakfast	0.4 ± 0.7
	22. I get sleepy during class outside of naptime	0.8 ± 0.9
	23. I fall asleep during class outside of naptime	0.2 ± 0.6
	24. I fall asleep if I sit still (i.e., watching TV, riding in the car)	0.8 ± 0.9

**Table 3:** Results of the confirmatory factor analysis.

Measure	Results	Good fit	Accepted fit
CMIN/DF	2.1	<2	<3
SRMR	0.0789	<0.05	<0.08
AGFI	0.805	>0.90	>0.85
CFI	0.845	>0.95	>0.90
RMSEA	0.071	<0.05	<0.08

CMIN/DF, chi-squared/degrees of freedom; SRMR, standardized root mean square residual; AGFI, adjusted goodness-of-fit index; CFI, comparative fit index; RMSEA, root mean square error of approximation.

## Discussion

This study is the first to verify the factor structure of the CASC-P using CFA in a general population of parents of junior high school students in Japan. The CASC-P showed satisfactory internal consistency (Cronbach's alpha: 0.771), similar to a previous study on the self-reported CASC in junior high school students (Cronbach's alpha = 0.786) (17). However, this study found that some items did not have a good fit.

In general, a modified model needs to remove the non-good-fit items and attempt the next model using CFA. However, these non-good-fit items were difficult for parents to grasp. Items 1 and 2 are activities for children, such as drinking caffeine and playing games or using the Internet before bedtime, and parents find it difficult to answer these for their children. According to a Chinese survey, over 80% of early adolescents (average age = 10.83) sleep in separate bedrooms from their parents (18). Furthermore, most children use digital devices such as smartphones before bedtime. Although children generally have better age-appropriate sleep in the presence of household rules and regular sleep-wake routines, sleep deficiency is more likely to occur when parents and children have electronic devices in their bedrooms (1). According to a questionnaire survey involving primary schools in Japan, the use of digital devices was linked to children's emotional and psychological development (19). Item 2, “I play video games, surf the Internet, or send texts ≤1 h before going to bed,” had the highest score in the current study, indicating that many parents took this issue seriously. However, the relationship between sleep problems and digital device use is complicated; it may be difficult for parents to report their children's use of devices with a single question.

Item 9, “I stop breathing while I sleep,” received a very low score in this study. However, it is an important issue that some children stop breathing while sleeping. The exact prevalence of sleep-disordered breathing (SDB) in children is unknown but may be as high as 11% (20). SDB encompasses a spectrum of sleep disorders, including primary snoring at the mild end and obstructive sleep apnea at the severe end. Screening with a brief questionnaire is necessary to determine the risk of SDB and carefully inspect children at risk for SDB (21). Although the factor loading for this item was low, this may be due to underrecognition by parents rather than a lack of clinical relevance. Parental awareness of SDB symptoms is often limited, especially when children sleep in separate rooms. Furthermore, SDB is known to be associated with serious consequences such as impaired neurocognitive development, behavioral problems, and enuresis (22). Therefore, despite the statistical weakness of item 9, we consider it clinically important to retain this item for screening purposes.

Item 16, “I urinate in my sleep,” was also marked “very rarely” in the current study; this item did not obtain a good fit by CFA. The CASC-P was designed to assess the weekly frequency of enuresis comorbidity with SDB. Hence, the nocturnal enuresis score was low. For a diagnosis of nocturnal enuresis, children older than 6 years of age should have one or more wetting episodes per month (23). Nocturnal enuresis is commonly observed in children with SDB, and adenotonsillar hypertrophy is a significant contributor to SDB; consequently, adenotonsillectomy is the mainstay treatment for SDB (24, 25). To data, several pediatric sleep questionnaires have been developed internationally, including the Children's Sleep Habits Questionnaire (CSHQ), and the Pediatric Sleep Questionnaire (PSQ), each with different focuses and lengths. The CASC-P offers a concise structure while covering a full range of sleep-related behaviors throughout the day and night, making it well-suited for large-scale screening.

The present study has several limitations. First, the original CASC has not itself undergone comprehensive validation. In this study, the validation approach employed was limited, relying exclusively on validity using CFA, and reliability was assessed using Cronbach's alpha. In addition to structural validity, key aspects such as content validity, test-retest reliability, and construct validity should be addressed. Second, the assessment relied on parental reports. Only parental data are limited for such reasons as self-report bias and over-estimation of the associations between variables. Third, we did not control for confounding factors such as family background, economic status, and academic performance. Family background and dynamics, including bed-sharing, are important for understanding children's sleep duration. Fourth, there is a potential for selection bias. The study participants were recruited from public junior high schools in suburban areas, which may have skewed the results. Fifth, the data for this study were collected in 2012. Since then, major societal changes, particularly the COVID-19 pandemic, have significantly affected adolescent routines, screen time, emotional well-being, and sleep habits (26). These changes may influence the current applicability of our findings, and future research should evaluate the CASC-P using updated post-pandemic data. Finally, while the overall model fit may be considered acceptable in Table 3, the results suggest there is potential for refinement in CFA.

## Conclusion

Our data indicate that the CASC-P is a suitable questionnaire for assessing parents' perspectives on adolescent sleep behavior. As sleep problems among early adolescents are common, to examine the reliability and validity of such measures are necessary. Future studies should include clinical populations, such as adolescents diagnosed with sleep disorders or receiving therapeutic interventions. Comparing CASC-P results across general and clinical samples would provide valuable information on the checklist's sensitivity and its ability to distinguish clinical cases from normative patterns.

## Data Availability

The original contributions presented in the study are included in the article/Supplementary Material, further inquiries can be directed to the corresponding author.
