# Supplementary material for: Development and field test of the child and adolescent sleep checklist for parents of community junior high school students
Source: Front Child Adolesc Psychiatry. 2025 Aug 12;4:1644128. doi: 10.3389/frcha.2025.1644128 (PMC12378057; doi:10.3389/frcha.2025.1644128)
Supplement: Supplementary file 1 [file Table1.docx]

**Supplementary Table 1A**

Standardized regression weights of the Child and Adolescent Sleep Checklist for parents (CASC-P)

| CASC 1 | <--- | Bedtime problems | 0.159 |
| --- | --- | --- | --- |
| CASC 2 | <--- | Bedtime problems | 0.186 |
| CASC 3 | <--- | Bedtime problems | 0.278 |
| CASC 4 | <--- | Bedtime problems | 0.878 |
| CASC 5 | <--- | Bedtime problems | 0.815 |
| CASC 6 | <--- | Bedtime problems | 0.512 |
| CASC 7 | <--- | Sleep breathing and unstable sleep | 0.441 |
| CASC 8 | <--- | Sleep breathing and unstable sleep | 0.421 |
| CASC 9 | <--- | Sleep breathing and unstable sleep | 0.072 |
| CASC 10 | <--- | Sleep breathing and unstable sleep | 0.673 |
| CASC 11 | <--- | Sleep breathing and unstable sleep | 0.612 |
| CASC 12 | <--- | Sleep breathing and unstable sleep | 0.304 |
| CASC 13 | <--- | Parasomnia and sleep movement | 0.478 |
| CASC 14 | <--- | Parasomnia and sleep movement | 0.464 |
| CASC 15 | <--- | Parasomnia and sleep movement | 0.489 |
| CASC 16 | <--- | Parasomnia and sleep movement | 0.199 |
| CASC 17 | <--- | Parasomnia and sleep movement | 0.466 |
| CASC 18 | <--- | Parasomnia and sleep movement | 0.472 |
| CASC 19 | <--- | Daytime problems | 0.479 |
| CASC 20 | <--- | Daytime problems | 0.663 |
| CASC 21 | <--- | Daytime problems | 0.170 |
| CASC 22 | <--- | Daytime problems | 0.610 |
| CASC 23 | <--- | Daytime problems | 0.437 |
| CASC 24 | <--- | Daytime problems | 0.455 |
